# Detecting Bacterial Biofilms Using Fluorescence Hyperspectral Imaging and Various Discriminant Analyses

**DOI:** 10.3390/s21062213

**Published:** 2021-03-22

**Authors:** Ahyeong Lee, Saetbyeol Park, Jinyoung Yoo, Jungsook Kang, Jongguk Lim, Youngwook Seo, Balgeum Kim, Giyoung Kim

**Affiliations:** Rural Development Administration, 310 Nongsaengmyeng-ro, Deokjin-gu, Jeonju 54875, Korea; lay117@korea.kr (A.L.); psb0911@korea.kr (S.P.); young3233@korea.kr (J.Y.); js72kang@korea.kr (J.K.); limjg@korea.kr (J.L.); yws25@korea.kr (Y.S.); bgkim8732@korea.kr (B.K.)

**Keywords:** *E. coli*, *S. typhimurium*, biofilm, hyperspectral imaging, discriminant analysis

## Abstract

Biofilms formed on the surface of agro-food processing facilities can cause food poisoning by providing an environment in which bacteria can be cultured. Therefore, hygiene management through initial detection is important. This study aimed to assess the feasibility of detecting *Escherichia coli* (*E. coli*) and *Salmonella typhimurium* (*S. typhimurium*) on the surface of food processing facilities by using fluorescence hyperspectral imaging. *E. coli* and *S. typhimurium* were cultured on high-density polyethylene and stainless steel coupons, which are the main materials used in food processing facilities. We obtained fluorescence hyperspectral images for the range of 420–730 nm by emitting UV light from a 365 nm UV light source. The images were used to perform discriminant analyses (linear discriminant analysis, *k*-nearest neighbor analysis, and partial-least squares discriminant analysis) to identify and classify coupons on which bacteria could be cultured. The discriminant performances of specificity and sensitivity for *E. coli* (1–4 log CFU·cm^−2^) and *S. typhimurium* (1–6 log CFU·cm^−2^) were over 90% for most machine learning models used, and the highest performances were generally obtained from the *k*-nearest neighbor (*k*-NN) model. The application of the learning model to the hyperspectral image confirmed that the biofilm detection was well performed. This result indicates the possibility of rapidly inspecting biofilms using fluorescence hyperspectral images.

## 1. Introduction

Despite the increasing social interest in safe agro-food, food poisoning occurs frequently. Food poisoning due to foodborne illnesses is one of the major public health problems worldwide [1]. The Centers for Disease Control and Prevention (CDC) estimates that food poisoning causes 48 million people to get sick, 128,000 to be hospitalized, and 3000 to die each year [2]. Although the cause of food poisoning varies, most food poisoning incidents are caused by ingestion of food contaminated with germs or viruses [3,4]. In the case of food contamination occurring in the process of food processing and distribution, cross-contamination is one of the main routes of contamination caused by the surface of germ-contaminated facilities, machinery, and containers and contaminated washing water [5,6].

Food safety accidents caused by cross-contamination in food processing facilities [7,8,9,10,11] or home kitchens [12,13,14] are related to biofilms found in a wide range of environments. Biofilms are composed of an extracellular polymeric substance, which comprises mostly polysaccharides secreted by microorganisms. Biofilms are firmly attached to the surface of living organisms (vegetables, meat, etc.) or non-living objects (stainless steel, plastics, etc.) [15]. Sessile bacteria attached to biofilms are known to withstand stress better than planktonic bacteria in stressful environments such as in the presence of antibiotics, disinfectants, high temperatures, light, and dryness [16,17]. Cross-contamination occurs when food passes through a surface contaminated with biofilms or when cells are separated from the biofilm structure through an aerosol derived from a contaminated food processing facility [1,11]. According to the US National Institutes of Health, about 80% of human infections are related to biofilms [18]. These biofilms cause serious hygiene problems and economic losses due to food spoilage, equipment damage, and food poisoning (microbiological infection). Therefore, this is an important issue for the food industry, including the agricultural and livestock production and processing industries, to tackle [19].

Soon [4] conducted a factor analysis through cases of food safety incidents and recalls of food safety accidents that occurred around the world during 2008–2018. He revealed that 40.11% of food safety hazards were caused by microbiological hazards, and the major hazards were *Listeria monocytogenes* (32.91%), *Salmonella spp.* (29.85%), and *Escherichia coli* (17.86%). Among them, *Escherichia coli* (*E. coli*) infection can occur by eating contaminated agricultural products (sprouts, spinach, lettuce, etc.) or undercooked meat and is highly likely to lead to hospitalization in disease outbreaks [2]. Food poisoning due to *E. coli* infection is associated with serotype O157:H7. In particular, Shiga toxin-producing *Escherichia coli* causes hemolytic uremic syndrome (also known as Hamburger’s disease), which leads to acute kidney damage; it is a fatal disease in children and the elderly [20]. *Salmonella typhimurium* (*S. typhimurium*) causes salmonellosis, which leads to acute and chronic enteritis symptoms such as diarrhea and abdominal pain [21]. The main sources of disease are contaminated poultry, meat, and eggs [22]. The CDC estimates that *Salmonella* causes approximately 1.2 million diseases and 450 deaths each year in the United States and reports that it is the most common cause of food poisoning in June, July, and August [2,23]. As noted in many studies, *E. coli* [11,24,25,26] and *S. typhimurium* [27,28,29,30,31,32] form a strong biofilm on the surface of non-living objects or living organisms. Cross-contamination in agro-food processing lines caused by biofilms is a serious concern in the food industry.

There is growing demand for technology that can quickly and easily detect biological contaminants to prevent cross-contamination [33,34]. Conventional biofilm testing techniques use swabs to collect samples of the suspected area. After that, the bacteria are cultured to check the presence of contamination using various methods such as contact plate system, microbial diagnostic platform like TEMPO^®^ (bioMerieux, Marcy-l’Etoile, France), and adenosine triphosphate (ATP) determination [35,36]. In this case, testing performance is degraded when we collect samples using swabs, and it takes a long time to cultivate bacteria. As an alternative, hyperspectral imaging (HSI) technology with rapid and non-destructive inspection characteristics is drawing attention [37]. HSI technology can not only identify the physical chemistry characteristics of a substance through spectroscopic analysis but also simultaneously obtain information about the spatial distribution of certain components through image analysis [38]. Since hyperspectral imaging has many independent variables, analysis methods that can reduce the number of independent variables have been used rather than using general multiple regression analysis [39,40,41,42,43]. Among them, partial least-squares regression (PLSR) and principal component analysis (PCA) are mainly used. PLSR analysis is suitable for regression modeling under the condition that the number of samples is less than the number of variables. PCA is suitable for classification modeling that reduces the number of independent variables through dimensional transformation [44]. Recently, there are increasing cases of applying various machine learning techniques [45,46] or artificial neural networks [47,48,49] to increase the performance of the model.

Many studies have demonstrated that HSI technology is a powerful tool for monitoring food safety incidents in relation to biofilms, which cause cross-contamination in the food industry [50,51,52,53,54]. Zhu [54] determined corn contaminated by aflatoxin mycotoxin over 100 ppb with 95.3% performance using fluorescence hyperspectral images. Jun [52] identified a biofilm over 7 log CFU·cm^−2^ formed by *E. coli* and *S. typhimurium* with a performance of 95% using the one-wavelength image and the ratio image of the fluorescence hyperspectral image of two wavelength bands. Lee [15] identified a biofilm over 1 log CFU·cm^−2^ formed by *E. coli* on a high-density polyethylene (HDPE) coupon. Lee used the single-wavelength method that Jun [52] used for detecting biofilm and machine learning techniques. Then Lee confirmed that the prediction performance of a biofilm by machine learning techniques is higher than that by the single-wavelength method.

This study was conducted to examine the feasibility of rapidly inspecting biofilms using HSI technology by expanding the targets of detection than the previous research [15] we did. First, we confirmed the fluorescence characteristics of *E. coli* and *S. typhimurium* using a microplate reader. Then, we obtained a biofilm fluorescence image between 420 and 730 nm for a 365 nm UV light source using a hyperspectral imaging device. The biofilm is formed by *E. coli* and *S. typhimurium* on the surface of HDPE and stainless steel (SS), which are the main materials in agro-food processing facilities. Consequently, we developed a biofilm discrimination model for hyperspectral images by applying various machine learning algorithms and compared their performances.

## 2. Materials and Methods

### 2.1. Fluorescence Characteristics of Food Poisoning Bacteria

To examine the fluorescence characteristics of food poisoning bacteria, strains of non-pathogenic *Escherichia coli* (*E. coli*, KCCM11234) and *Salmonella typhimurium* (*S. typhimurium*, KCCM12041) were obtained from the Korean Culture Center of Microorganisms. All reagents and media were sterilized at 121 °C for 15 min using an autoclave (MLS–2420; SANYO, Tokyo, Japan) before use. Each strain was individually grown in tryptic soy broth (TSB; BD, Franklin Lakes, NJ, USA) at 36 °C for 24 h for activation. The cultures were transferred onto the surface of a tryptic soy agar (TSA; BD, Franklin Lakes, NJ, USA) plate by loop and incubated at 36 °C for 24 h. Single colonies, which were formed after incubation, were collected from the plates and were suspended in 0.1 M phosphate-buffered saline (PBS) solution in a microtube. The suspension was centrifuged at 8000 rpm for 3 min (Eppendorf centrifuge 5804 R; Eppendorf, Hamburg, Germany). Then, the supernatant was removed using a pipette, and the pellets were resuspended in 0.1 M PBS solution. The washing step was performed three times. The optical density (OD) of the cell suspension was measured at 600 nm using a microplate reader (Infinite M1000; Tecan, Männedorf, Switzerland), and the final concentration was adjusted to 10^9^ CFU·mL^−1^ (OD 0.1 and 100 µL). Then, each strain was serially diluted tenfold from 10^6^ to 10^1^ with 0.1 M PBS solution. The 0.1 M PBS solution was used as a control group. Each cell suspension with the adjusted number of *E. coli* and *S. typhimurium* cells was placed in a 96-well plate. Fluorescence emission intensity from 400 to 800 nm bands was acquired for excitation light from 350 to 400 nm bands at 5 nm intervals using a microplate reader.

### 2.2. Bacterial Biofilm Formation

**Figure 1:**
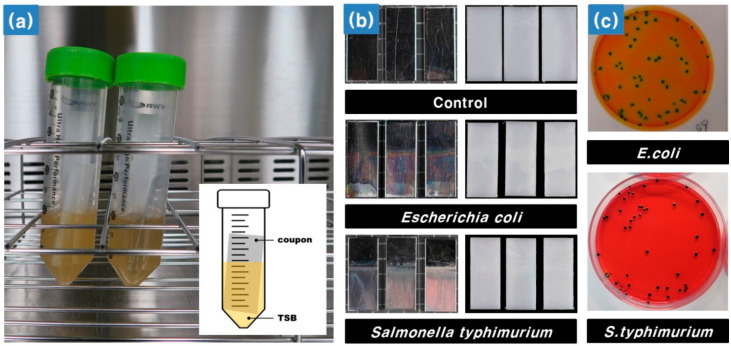
(**a**) Bacterial biofilm formation using a conical tube, (**b**) stainless steel (SS) coupons (left) and high-density polyethylene (HDPE) coupons (right), and (**c**) cultivation of *Escherichia coli* (*E. coli*) and *Salmonella typhimurium* (*S. typhimurium*).

The biofilm was formed by using the non-pathogenic *E. coli* and *S. typhimurium* strains that were previously obtained. All HDPE and SS coupons (20 × 50 × 1 ${\mathrm{mm}}^{3}$) for the formation of biofilms were washed with an ultrasonic cleaner (WUC-A, DAIHAN Ultrasonic cleaner; Wonju, Korea), which was sterilized at 121 °C for 15 min in an autoclave (MLS–2420; SANYO, Tokyo, Japan) and then completely dried before use. The OD of the cell suspension was measured at 600 nm using a microplate reader, and 100 μL of the cell suspension was adjusted to an OD value of 0.1. To achieve biofilm formation on the surface of HDPE and SS coupons, the cell suspension was inoculated in a 50 mL conical tube containing 15 mL of TSB (approximately 10^2^ CFU·mL^−^^1^), and coupons were placed in a conical tube and incubated at 36 °C for seven days, as shown in Figure 1a. Every 24 h interval, the culture medium of the conical tube was removed and 0.1 M PBS solution was added and washed twice with gentle stirring. Then, 15 mL of sterilized TSB medium was added to supply nutrients for biofilm formation. For the control, the process proceeded under the same conditions, but the bacteria were not inoculated into the TSB medium.

After biofilm incubation, each coupon was rinsed three times with distilled water using a pipette to reduce interference from other substances, such as the medium and loosely attached bacteria cells. Coupon surfaces were dried completely on a sterile workbench, from which hyperspectral images were acquired, as shown in Figure 1b. To enumerate attached cells, the biofilms formed on the surface of the coupon were carefully detached with a cell scraper. Detached biofilms were transferred into 0.1 M PBS solution and diluted sequentially. The diluted culture medium was incubated at 36 °C for 24–48 h. The degree of biofilm formation on the surface of coupons was determined by counting the number of colonies using the standard plate count (SPC) method and the dry-film method (Figure 1c). To determine the number of adherent cells, the SPC method was performed with plate count agar (Plate Count Agar; Becton Dickinson and Company, Franklin Lakes, NJ, USA), and the dry-film method used a rapid-dry film (3M Petri-film *E. coli*/coliform count plates; 3M, St. Paul, MN, USA) for *E. coli*. In the case of *S. typhimurium*, the cell suspension was streaked onto XLT4 selective media (Xylose-Lysine-Tergitol 4 agar; Becton Dickinson and Company, Sparks, MD, USA).

### 2.3. Hyperspectral Imaging System

**Figure 2:**
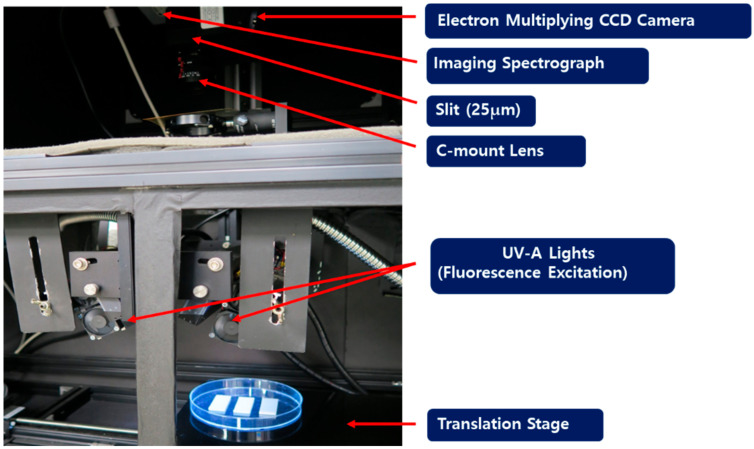
The ultraviolet fluorescence hyperspectral imaging system.

Figure 2 shows a fluorescence hyperspectral imaging system using ultraviolet (UV) excitation light. This system was composed of a highly sensitive electron-multiplying charge-coupled device (EMCCD, MegaLuca; Andor Technology Inc., Belfast, Northern Ireland) for obtaining hyperspectral images. The EMCCD camera consisted of 8 µm × 8 µm pixels and received a 14-bit digital image at a rate of 12.5 MHz. The EMCCD camera was thermo-electrically cooled to a temperature of −20 °C using a two-stage Peltier device. The imaging spectrograph (VNIR Hyperspec; HeadwallPhotonics Inc., Fitchburg, MA, USA) and a Schneider–Kreuznach Xenoplan 1.4/23 C-mount lens (f/1.9 35 mm Compact Lens; Schneider Optics, Hauppauge, NY, USA) were positioned in front of the EMCCD. The light source was a 365 nm UV beam (model XX–15A 365 nm; Spectronics Corp., Westbury, NY, USA), and the motorized sample stand was driven by a linear motor (XSlide; Velmax Inc., Bloomfield, NY, USA). The field of view of an image is limited by the size of the slit, which was 25 µm in this study. The fluorescence generated from the sample by the UV light source passed through the lens and slit of the imaging spectrograph. Then, the line scan image acquired through the slit was spectroscopically irradiated onto the EMCCD surface. Consequently, each line scan image was collected with spatial information horizontally and spectral information vertically.

### 2.4. Acquisition of Hyperspectral Fluorescence Images and Spectra

To investigate the possibility of biofilm detection using hyperspectral images, 7-day-passed coupons after inoculation of *E. coli* and *S. typhimurium* were used in each experiment: 15 HDPE coupons and 15 SS coupons were used as the test group contained in the culture medium inoculated with bacteria, while 9 HDPE coupons and 9 SS coupons were used as the control group treated in the culture medium without bacteria. Hyperspectral images were acquired for both sides (front and back) of the coupons, and 96 hyperspectral images were obtained in the end. In the case of *S. typhimurium*, 96 hyperspectral images were acquired in the same manner from the third to the fifth day after inoculation of *S. typhimurium*. There was a difference in the culture rates of *E. coli* and *S. typhimurium* even after adjusting for the initial number of cultured bacteria.

For each sample, we obtained hyperspectral fluorescence images, dark reference images, and white reference images. The fluorescence hyperspectral images were acquired by the line scanning method, with 340 lines and 1 mm intervals for exposure times of 100 ms using a UV beam. The hyperspectral image included a spatial resolution of 310 × 502 pixels and contained 420–730 nm wavelength images, which were equally divided into 65 bands, with a waveband interval of 4.8 nm. The hyperspectral image was averaged after 4 repeated measurements for the same location to remove the noise.

Dark reference plate images for device noise compensation were measured by blocking the light using the cover of the camera lens. Fluorescence reference plate images were measured using a plate where the fluorescence was uniformly displayed. The white reference plate images were measured by a premium white inkjet paper (Union Camp Co.) exhibiting uniform blue fluorescence [55]. Fluorescence hyperspectral images were transformed for a total of 65 bands using Equation (1):

(1) $$I_{fluorescence}\;\left(i\right)\;=\;\frac{I_{r}\;\left(i\right)-\;I_{d}\;\left(i\right)}{I_{f}\;\left(i\right)-I_{d}\;\left(i\right)}$$

where $I_{fluorescence}$ is the corrected relative fluorescence image, $I_{r}$ is the raw hyperspectral fluorescence image, $I_{f}$ is the hyperspectral white reference image, and $I_{d}$ is the hyperspectral dark reference image at the *i*-th wavelength. Before analyzing the hyperspectral fluorescence image of the biofilm, it was preprocessed by a normalization method to remove the effects of non-uniformity of the light source and the electrical noise signal of the hyperspectral imaging equipment. Then, pixel and average fluorescence spectra were extracted from the modified fluorescence hyperspectral image.

### 2.5. Biofilm Detection Algorithm

**Figure 3:**
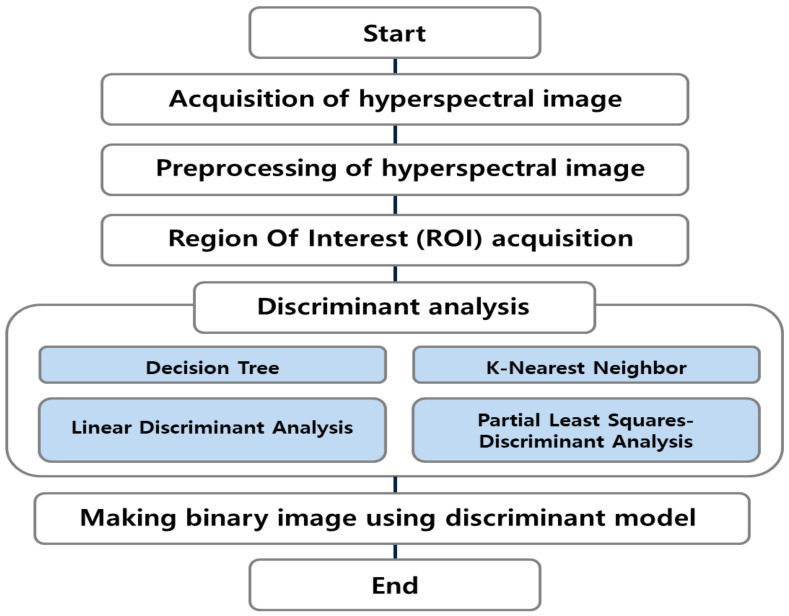
Flowchart of detecting bacterial biofilms using discriminant analyses.

Figure 3 shows a flowchart of the biofilm detection algorithm using hyperspectral imaging technology. As a preprocessing step, the original sample fluorescence hyperspectral images were corrected using dark and white reference images. A region of interest (ROI) of the spectra was extracted for the HDPE and SS coupon regions of the test group and the control group from the calibrated hyperspectral images. For the next step, the extracted spectra were discriminated and analyzed through various discriminant analyses. Discriminant analyses included decision trees (DTs), *k*-nearest neighbor (*k*-NN) analysis, linear discriminant analysis (LDA), and partial least-squares discriminant analysis (PLS-DA).

#### 2.5.1. Decision Tree

A DT predicts a class by plotting decision rules in a tree structure and classifying samples into several smaller groups. It is a top-down approach, where classes are divided by the partitioning rule until the stop criterion is met [56]. A DT is a popular supervised learning model for classification and regression because of its easy interpretation. However, it is easy to over-fit the training data, so tuning hyper-parameters (e.g., partitioning rule and stop criteria) is important.

#### 2.5.2. k-Nearest Neighbor

The *k*-NN classifies samples into the closest class based on the distance between samples in the feature space. It is named in that it predicts values from *k* neighbors [57]. The distance between samples is measured through the Euclidean distance. The Euclidean distance between the datum x and y is calculated using Equation (2):

(2) $$d\left(x,y\right)=\;\sqrt{{\left(x_{1}-y_{1}\right)}^{2}+{\left(x_{2}-y_{2}\right)}^{2}+\cdots +{\left(x_{n}-y_{n}\right)}^{2}}$$

where d represents the distance and n is the number of features. In the *k*-NN for classification, the input data are predicted as the largest number of class of the closest *k* data. As the value of *k* increases, the effect of noise can decrease, but the boundary between classes becomes unclear. Therefore, a process of finding proper *k*-value through repeated experiments is required [58].

#### 2.5.3. Linear Discriminant Analysis

LDA reduces the dimension of feature vectors by maximizing the ratio of variance between classes and within classes [59,60,61]. It means that LDA finds the optimal transformation matrix (w) that maximizes the criterion function $W_{lda}$, which is the ratio of the within-class scatter ($S_{w}$) and between-class scatter ($S_{B}$) like Equations (3)–(5):

(3) $$W_{lda}=\frac{\left|W^{T}S_{B}W\right|}{\left|W^{T}S_{W}W\right|}$$

(4) $$S_{w}=\overset{n}{\underset{i=1}{\sum }}\underset{x\in n_{i}}{\sum }\left(x-m_{i}\right){\left(x-m_{i}\right)}^{T}$$

(5) $$S_{B}=\overset{n}{\underset{i=1}{\sum }}k_{i}\left(m_{i}-m\right){\left(m_{i}-m\right)}^{T}$$

where *n* is the number of classes, $n_{i}$ is a set of data belonging to the *i*-th class, $m_{i}$ is the mean of the *i*-th class, and $k_{i}$ is the size of $n_{i}$.

#### 2.5.4. Partial Least Squares Discriminant Analysis

PLS-DA is an analysis technique based on PLSR that classifies predicted regression models using threshold values. Although PLS-DA and PLSR are the same analysis methods, PLSR uses continuous dependent variables (e.g., spectra) to develop and predict regression models, and PLS-DA is a variant version for categorical prediction models [62]. PLSR model is calculated using Equations (6) and (7):

(6) $$X\;=\;{TP}^{T}+E$$

(7) $$Y\;=\;{UQ}^{T}+F$$

where n is the number of samples, p is the number of variables, $X\;=\;\left(n\times p\right)$ matrix, $Y\;=\;\left(n\times 1\right)$ matrix, T and U are $\left(n\times p\right)$ score matrices of latent vectors, P and Q are matrices of loading, and E and F are the error terms (residuals). PLS-DA is performed by applying the score obtained through PLSR to discriminant analysis.

#### 2.5.5. Biofilm Detecting Performance

The development of an *E. coli* and *S. typhimurium* biofilm prediction model and detection of biofilm regions through discriminant analyses were performed using the open statistical software R (ver. 2019; R Foundation, Vienna, Austria) and the commercial software MATLAB (ver. 2018; MathWorks Inc., Matick, MA, USA). To develop a biofilm detection model, 80% of the spectra extracted from the hyperspectral images were used to develop a prediction model, and the remaining 20% of the spectra were used to verify the biofilm prediction performance. Fivefold cross-validation was performed to prevent over-fitting, and the prediction performance was calculated using Equations (8) and (9):

(8) $$\mathrm{Control}\;\mathrm{group}\;\mathrm{performance}\;\left(\mathrm{Specificity}\right)=\frac{TN}{TN+FP}$$

(9) $$\mathrm{Experimental}\;\mathrm{group}\;\mathrm{performance}\;\left(\mathrm{Sensitivity}\right)=\frac{TP}{TP+FN}$$

where TP (true positive) is the frequency of accurately predicting the area where the biofilm was formed, FP (false positive) is the frequency of erroneously predicting the area where the biofilm was not formed, TN (true negative) is the frequency of accurately predicting the region where the biofilm was not formed, and FN (false negative) is the frequency of erroneous prediction of the region where the biofilm was formed.

Additionally, the receiver operating characteristics (ROC) curve was drawn and the area under the curve (AUC) was calculated for choosing the best model. The ROC curve is the plot with the true-positive rate against the false-positive rate, and yjr AUC is the area under the ROC curve. After selecting the model with the highest performance, the biofilm detection result was validated by applying the model to hyperspectral images that were not used for model development.

## 3. Results and Discussion

### 3.1. Fluorescence Characteristics of Food Poisoning Bacteria

**Figure 4:**
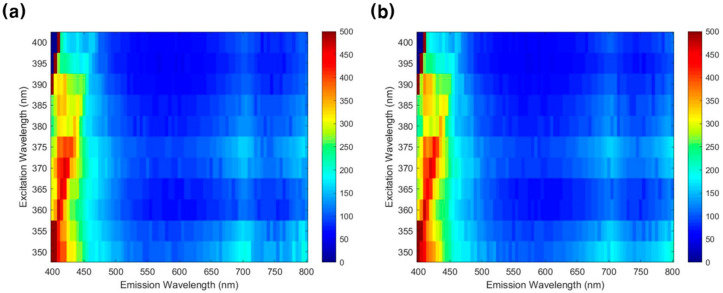
Fluorescence emission results from 400 to 800 nm bands for the excitation light from 350 to 400 nm bands: (**a**) *E. coli* and (**b**) *S. typhimurium*.

Figure 4 shows fluorescence emission spectra obtained by the microplate from 400 to 800 nm bands for excitation light from 350 to 400 nm bands. Figure 4a,b is the fluorescence emission spectra of *E. coli* and *S. typhimurium,* respectively. It can be seen that both *E. coli* and *S. typhimurium* cultures exhibited a high fluorescence expression intensity in 400–450 nm wavelength bands.

**Figure 5:**
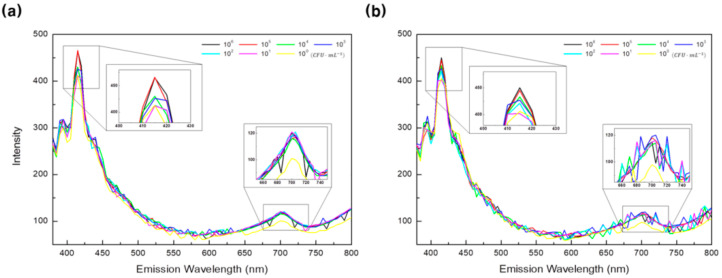
Fluorescence emission spectra for six concentrations (10^5^, 10^4^, 10^3^, 10^2^, 10^1^, and 10^0^ CFU·mL^−1^) of food poisoning bacteria for 365 nm excitation light: (**a**) *E. coli* and (**b**) *S. typhimurium*.

Figure 5 shows the fluorescence emission spectra for each concentration of food poisoning bacteria for 365 nm excitation light. Figure 5a,b shows the fluorescence emission spectra of *E. coli* and *S. typhimurium* concentrations controlled at 10^5^, 10^4^, 10^3^, 10^2^, 10^1^, and 10^0^ (PBS) CFU·mL^−1^, respectively. It can be seen that the fluorescence intensity is high in the 400–450 nm wavelength bands and around 700 nm bands. It was also confirmed that both *E. coli* and *S. typhimurium* showed high fluorescence intensity in the 415 nm wavelength band according to the bacterial concentration. In the case of the band around 700 nm, the bacterial culture medium showed higher fluorescence intensity than the PBS solution, but there was no difference in fluorescence intensity according to the bacterial concentration.

### 3.2. Food Poisoning Bacteria Biofilm Formation

As a result of measuring the number of bacteria using the standard plate count method and dry-film method, E. coli was successfully cultured on 16 pieces of HDPE coupons and 26 pieces of SS coupons, while *S. typhimurium* was cultured on 23 pieces of HDPE coupons and 26 pieces of SS coupons.

**Figure 6:**
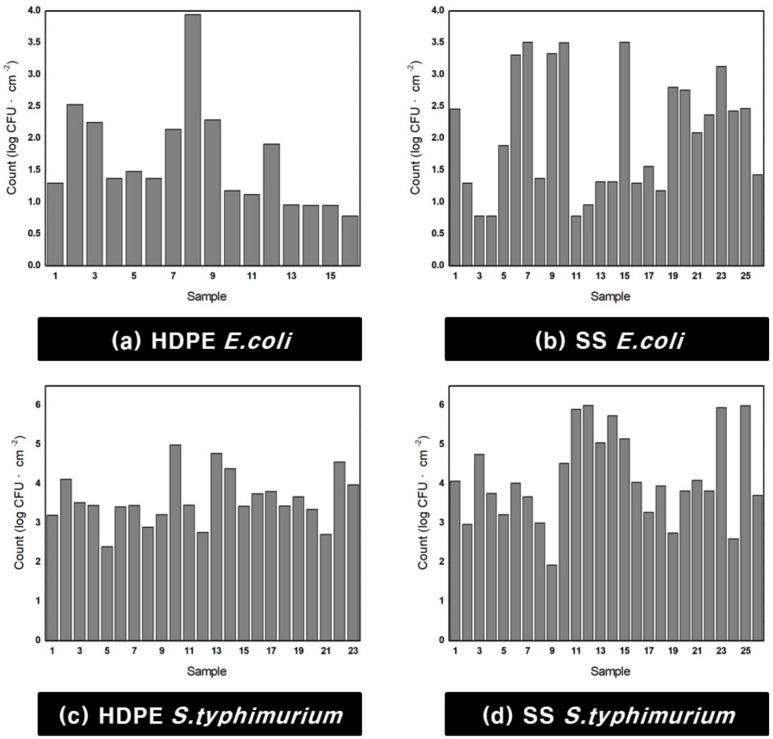
Cultivated *E. coli* and *S. typhimurium* count graph: (**a**) *E. coli* cultivated on high-density polyethylene (HDPE), (**b**) *E. coli* cultivated on stainless steel (SS), (**c**) *S. typhimurium* cultivated on HDPE, and (**d**) *S. typhimurium* cultivated on SS.

Figure 6 shows the number of bacteria measured for each culture. The number of *E. coli* per HDPE coupon ranged from 0.78 to 3.94 log CFU·cm^−2^, whereas the number of *E. coli* per SS coupon ranged from 0.78 to 3.51 log CFU·cm^−2^. In the case of *S. typhimurium*, 2.4 to 4.99 log CFU·cm^−2^ bacteria were formed on HDPE coupons and 1.93 to 6 log CFU·cm^−2^ bacteria were formed on SS coupons.

**Figure 7:**
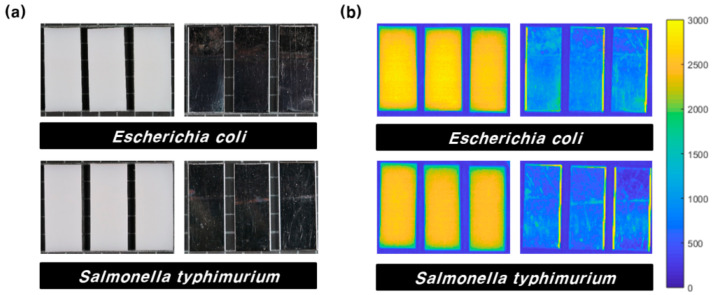
HDPE and SS coupons of bacterial biofilms: (**a**) RGB images and (**b**) hyperspectral images of the 551.8 nm band.

Figure 7a,b shows the RGB hyperspectral images of the 551.8 nm band for the test group coupons. In the case of RGB images, areas were not visually separated based on whether the biofilm was formed or not. In the case of hyperspectral images, the intensity difference was not significant, but it is difficult to distinguish a biofilm region using only one wavelength image.

Spectrum extraction from the hyperspectral image was performed using the area contained in the medium. A total of 183,212 spectra were extracted for use in model development. In the case of *E. coli*, 17,185 spectra were extracted from 10 of 16 HDPE coupons and 25,926 spectra were extracted from 15 of 26 SS coupons for model development. A total of 31,921 spectra were extracted from 18 of 23 HDPE coupons and 34,600 spectra were extracted from 18 of 26 coupons for the *S. typhimurium* model. As a control group, 19,662 and 15,709 spectra were extracted from 12 HDPE and 12 SS coupons in the case of the *E. coli* model, respectively. In the case of the *S. typhimurium* model, 19,053 and 19,156 spectra were extracted from 12 HDPE and 12 SS coupons, respectively. Consequently, 36,847 spectra were used to develop a model to detect *E. coli* on HDPE coupons, and 41,635 spectra were used to detect *E. coli* on SS coupons. In the case of *S. typhimurium,* 50,974 and 53,756 spectra were used for detection on HDPE and SS coupons, respectively.

### 3.3. Biofilm Detection Model

**Figure 8:**
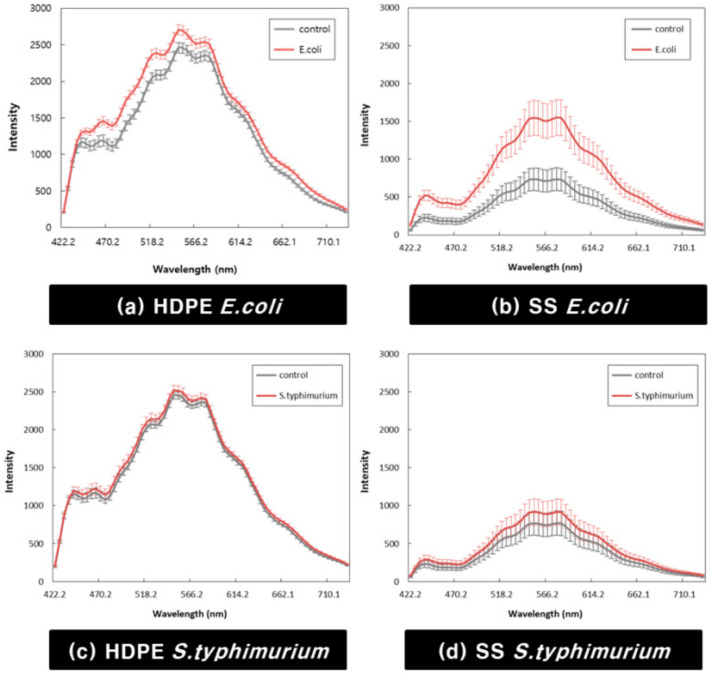
Average spectra of extracted pixel spectra from hyperspectral images: (**a**) *E. coli* cultivated on HDPE, (**b**) *E. coli* cultivated on SS, (**c**) *S. typhimurium* cultivated on HDPE, and (**d**) *S. typhimurium* cultivated on SS.

Figure 8 shows the average value of the extracted pixel spectra to develop a biofilm detection model. Figure 8a,b shows the average spectrum of *E. coli* cultured on HDPE and SS coupons, and Figure 8c,d shows the average spectrum of *S. typhimurium* cultured on HDPE and SS coupons. Both HDPE and SS coupons showed the highest fluorescence signal near 550 nm, and the fluorescence signal of SS coupons was low in all wavelength regions compared to HDPE coupons. It can be seen that the fluorescence signal intensity of the region where the biofilm is formed appears higher than that of the control region for all wavelength ranges. However, the wavelength bands could not indicate a distinct difference between the experimental group and the control group, except for the *E. coli* biofilm formed on SS coupons. Moreover, the spectral deviation was severe for each pixel, so it was be difficult to distinguish the two groups with only part of the wavelengths.

The biofilm detection model was developed by applying discriminant analyses using multiple wavelength bands. The DT, *k*-NN, LDA, and PLS-DA methods were used, which are widely used in discriminant analysis. After dividing the total amount of data in the 80:20 ratio, the model was trained using 80% of the data and the performance of the model was verified using the remaining 20% of the data. Fivefold cross-validation was performed, and average performances of specificity and sensitivity were calculated. Table 1 shows the results of discriminant analyses after classifying the test group’s spectrum by 1 and the control group’s spectrum by 0.

In general, discriminant model performances of specificity and sensitivity were higher than 90%, except the *S. typhimurium* model for SS coupons. The performances of the detection model for the biofilm formed on HDPE coupons was higher than that of the biofilm formed on SS coupons. It is estimated that the edge part of SS coupons reflected fluorescence, which made model learning difficult. In addition, it was found that the models used on *E. coli* had higher detection performances than those used on *S. typhimurium*. In particular, the *k*-NN model average specificity in the test group was 99.98% and 97.86% and the average sensitivity was 100% and 94.96% for the HDPE coupons of *E. coli* and *S. typhimurium*, respectively. The average specificity of the *k*-NN model in the test group was 96.25% and 85.35% and the average sensitivity was 92.61% and 39.47% for the SS coupons of *E. coli* and *S. typhimurium*, respectively, showing higher classification performances than other discriminant analyses. However, in some cases, the test group showed better performance than the *k*-NN model. In the case of the *S. typhimurium* model for HDPE coupons, the average sensitivity of LDA in the test group was 96.36%, and for the *E. coli* model for SS coupons, the average sensitivity was 92.83%. In the case of the *S. typhimurium* model for SS coupons, the average specificity of the DT analysis in the test group was 97.98%. Nevertheless, the *k*-NN model showed the highest performance under many conditions.

**Figure 9:**
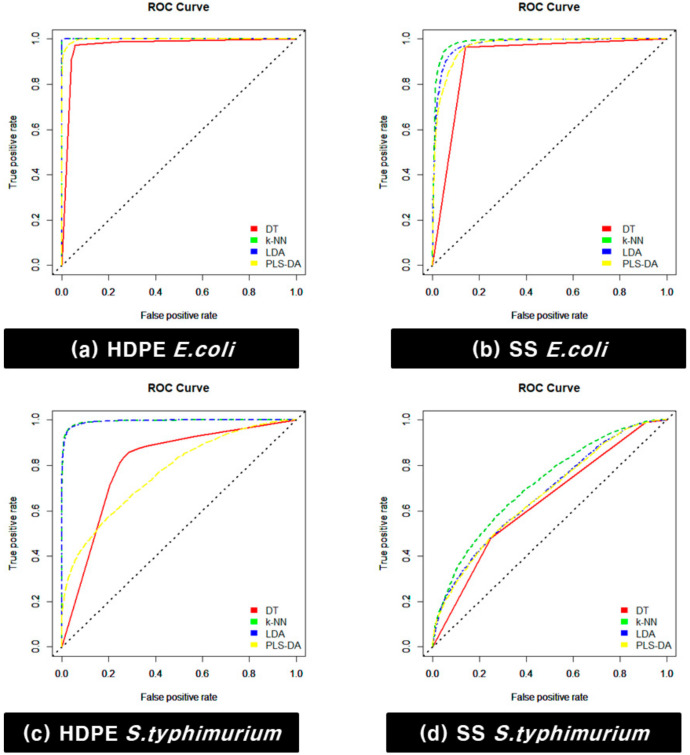
Receiver operating characteristics (ROC) curve of the discriminant model: (**a**) *E. coli* cultivated on HDPE, (**b**) *E. coli* cultivated on SS, (**c**) *S. typhimurium* cultivated on HDPE, and (**d**) *S. typhimurium* cultivated on SS.

Then, the ROC curve was drawn (Figure 9), and the AUC was calculated for choosing the best model. In the case of the *E. coli* model for HDPE coupons, the AUC of the DT analysis in the test group was 0.968, that of the *k*-NN analysis was 1, that of LDA was 1, and that of PLS-DA was 0.997. In the case of the *S. typhimurium* model for HDPE coupons, the AUC of the DT analysis in the test group was 0.808, that of the *k*-NN analysis was 0.994, that of LDA was 0.994, and that of PLS-DA was 0.769. In the case of the *E. coli* model for SS coupons, the AUC of the DT analysis in the test group was 0.911, that of the *k*-NN analysis was 0.984, that of LDA was 0.975, and that of PLS-DA was 0.967. In the case of the *S. typhimurium* model for SS coupons, the AUC of the DT analysis in the test group was 0.636, that of the *k*-NN analysis was 0.715, that of LDA was 0.671, and that of PLS-DA was 0.667. Therefore, the *k*-NN algorithm is suitable for detecting *E. coli* and *S. typhimurium* biofilms.

### 3.4. Food Poisoning Bacteria Biofilm Detection Result

**Figure 10:**
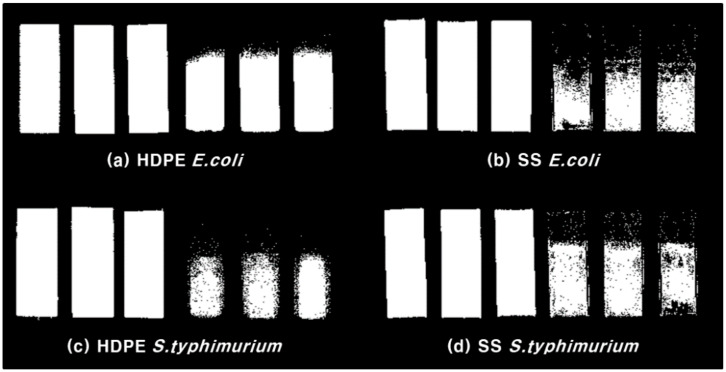
Region of interest of coupons (left) and bacterial biofilm detection result (right): (**a**) *E. coli* cultivated on HDPE, (**b**) *E. coli* cultivated on SS, (**c**) *S. typhimurium* cultivated on HDPE, and (**d**) *S. typhimurium* cultivated on SS.

Among discriminant analyses, the *k*-NN model, which showed the highest detection performance, was applied to all pixels constituting the hyperspectral image to predict the presence of a biofilm. First, the coupon region area and background area were binarized by setting the ROI in the image. Then, the *k*-NN model was applied to the coupon region area. Figure 10 shows the binarization result of bacterial detection. It was confirmed that the biofilm area, which was difficult to determine with the naked eye, was successfully detected. Jun [52] detected the *E. coli* biofilm on the surface of SS coupons using a short wavelength, and the minimum detection limit of the *E. coli* biofilm was 7 log CFU·cm^−2^. In addition, the same research group detected the *E. coli* biofilm on the surface of HDPE coupons using a short wavelength and a ratio of two wavelength images in the hyperspectral image. The minimum detection limit of *E. coli* was reported to be 7.56 log CFU·cm^−2^. Lee [15] detected the *E. coli* biofilm formed on the surface of HDPE coupons using multiple wavelengths and confirmed that the *k*-NN algorithm had the highest detection performance. In this study, biofilms of *E. coli* and *S. typhimurium* were detected using discriminant analyses and the *k*-NN model had the highest detection performance, which agrees with the results reported by Lee [15]. In addition, it was confirmed that the *E. coli* biofilm formed at a concentration of 0.78 log CFU·cm^−2^ and *S. typhimurium* biofilm formed at a concentration of 1.93 log CFU·cm^−2^ could be detected. Using the *k*-NN algorithm, the minimum detection limit was lowered to almost one-tenth of that in previous studies. Recently, as sensor technology has rapidly developed, miniaturization of the hyperspectral imaging system has also progressed. Currently, our research was conducted in a laboratory environment, so there is a limitation that learning models cannot be used directly in the field. However, if a portable hyperspectral system that is capable of implementing our learning model is made, real-time biofilm detection would be possible in the field.

## 4. Conclusions

This study was conducted to determine whether biofilms, which are the main cause of cross-contamination of bacteria in agro-food processing facilities, can be quickly inspected using hyperspectral imaging technology and various discriminant analysis techniques. Biofilms were formed by *E. coli* and *S. typhimurium* on the surface of HDPE and SS coupons, which are used as main materials in agro-food processing facilities.

To examine the fluorescence characteristics of food poisoning bacteria, the fluorescence emission intensity of *E. coli* and *S. typhimurium* from 400 to 800 nm bands was acquired for excitation light from 350 to 400 nm bands at 5 nm intervals using a microplate reader. As a result, high fluorescence intensity was confirmed according to the bacterial concentration in the 400–450 nm wavelength bands, and fluorescence expression was also confirmed in the 700 nm band.

Hyperspectral fluorescence images of a 365 nm UV light source in the wavelength range of 420 to 730 nm were acquired using a line scan apparatus. The average spectra of the samples were extracted from the corrected hyperspectral image data. Extracted spectra were analyzed by various discriminant analyses such as DTs, *k*-NN, LDA, and PLS-DA. The *k*-NN algorithm predicted the biofilm region with a high performance of 90% or more. The minimum detection limit of the detected biofilm was 0.78 log CFU·cm^−2^ and 1.93 log CFU·cm^−2^ for *E. coli* and *S. typhimurium*, respectively. The biofilm detection model using the *k*-NN algorithm was applied to all pixels of the hyperspectral images, and it was found that the biofilm region could be accurately detected. The possibility of real-time biofilm detection in the field using hyperspectral images was confirmed.

In the case of discriminant analysis, there is a disadvantage that the data volume used for model development is large and the model training time is long. These issues can be solved using an optimization technique by selecting wavelength bands essential for model development. In addition, further research is required to secure more samples to reduce the false detection rate.

## Figures and Tables

**Table 1:** Discriminant model performance.

				Performance (%)											
				1		2		3		4		5		Average	
				spe	sen	spe	sen	spe	sen	spe	sen	spe	sen	spe	sen
HDPE	*E. coli*	DT	*Train*	97.05	94.09	91.07	95.89	96.64	96.53	94.33	95.04	94.23	95.07	94.66	95.32
			*Test*	97.06	94.86	91.33	95.79	96.52	96.04	93.53	94.88	94.03	94.61	94.49	95.24
		*k*-NN	*Train*	99.99	100	100	100	99.99	100	99.99	100	99.99	100	**99.99**	**100**
			*Test*	99.97	100	100	100	100	100	99.94	100	100	100	**99.98**	**100**
		LDA	*Train*	99.82	100	99.81	100	99.78	99.99	99.79	99.99	99.75	100	99.79	**100**
			*Test*	99.65	99.97	99.71	100	99.85	99.97	99.8	100	99.91	100	99.78	99.99
		PLS-DA	*Train*	96.76	98	96.67	97.97	96.8	98.02	96.78	97.89	96.63	97.61	96.73	97.90
			*Test*	96.74	98.19	96.66	97.72	96.67	97.78	96.59	97.91	97.06	97.53	96.74	97.83
HDPE	*S. typhimurium*	DT	*Train*	85.4	76.41	84.46	78.66	87.63	69.57	84.81	76.78	86.69	70.64	85.80	74.41
			*Test*	84.07	75.89	83.71	76.79	87.48	69.84	85.18	75.89	85.85	70.22	85.26	73.73
		*k*-NN	*Train*	98.9	97.24	98.86	97.36	98.94	97.21	98.97	97.21	98.85	97.22	**98.90**	**97.25**
			*Test*	97.85	94.83	97.78	94.7	97.64	95.13	98.19	95.29	97.84	94.85	**97.86**	94.96
		LDA	*Train*	96.47	96.26	96.52	96.46	96.61	96.29	96.5	96.27	96.46	96.42	96.51	96.34
			*Test*	96.65	96.69	96.47	95.96	96.3	96.31	96.5	96.68	96.53	96.15	96.49	**96.36**
		PLS-DA	*Train*	87.17	44.95	86.92	45.42	87.15	45.11	87.01	44.68	87.23	45.19	87.10	45.07
			*Test*	87.13	45.25	86.69	46.02	87.1	44.78	88.25	44.76	86.47	44.41	87.13	45.04
SS	*E. coli*	DT	*Train*	95.93	85.35	96.31	84.88	96.24	84.77	95.84	85.33	96.29	84.92	96.12	85.05
			*Test*	95.55	85.81	96.18	84.26	96.46	84.73	95.9	85.88	96.24	84.13	96.07	84.96
		*k*-NN	*Train*	98.41	93.43	98.32	93.31	98.38	93.51	98.35	93.37	98.67	94.31	**98.43**	**93.59**
			*Test*	97.21	92.12	97.51	91.19	97.34	90.84	91.85	97.67	97.35	91.21	**96.25**	92.61
		LDA	*Train*	92.15	92.9	92.09	93.2	92.01	93	92	92.87	92.19	92.93	92.09	92.98
			*Test*	91.9	93.16	92.46	91.9	92.47	92.62	91.86	93.52	91.94	92.95	92.13	**92.83**
		PLS-DA	*Train*	91.66	89.2	91.58	89.47	91.54	89.41	91.53	89.41	91.43	89.44	91.55	89.39
			*Test*	90.14	91.39	90.96	89.26	92.05	89.06	91.39	89.48	92.01	88.99	91.31	89.64
SS	*S. typhimurium*	DT	*Train*	99.19	9.17	92.82	23.26	99.18	9.2	99.41	8.43	99.45	8.41	**98.01**	11.69
			*Test*	99.22	9.17	92.73	21.72	99.28	9.02	99.4	8.44	99.25	8.51	**97.98**	11.37
		*k*-NN	*Train*	94.37	58.83	94.27	58.26	94.58	58.12	94.58	58.2	94.78	58.41	94.52	**58.36**
			*Test*	85.65	38.29	85.67	40.77	84.42	40.36	85.26	38.41	85.75	39.5	85.35	**39.47**
		LDA	*Train*	91.05	26.42	90.76	26.84	91.02	26.74	91.15	26.31	91.12	26	91.02	26.46
			*Test*	91.4	26.12	90.83	26.02	90.86	25.46	90.9	26.86	91.07	27.47	91.01	26.39
		PLS-DA	*Train*	93.89	20.81	93.64	21.27	93.93	20.82	93.93	20.88	94.02	20.55	93.88	20.87
			*Test*	94.36	20.65	93.69	20.38	93.77	20.87	93.53	20.93	94.01	21.33	93.87	20.83

## Data Availability

Data sharing not applicable.
